# Enhancing diagnostic tools for vasopressin deficiency: insights from a single-center cohort study

**DOI:** 10.1007/s11102-025-01538-9

**Published:** 2025-05-29

**Authors:** Alessandro Mondin, Giulia Bovo, Giorgia Antonelli, Diego Faggian, Pierluigi Mazzeo, Alessandro Bavaresco, Filippo Ceccato, Mattia Barbot

**Affiliations:** 1 Department of Medicine DIMED, University of Padova, Via Ospedale Civile 105, Padova, 35128 Italy; 2 Endocrinology Unit, University Hospital of Padova, Padova, 35128 Italy; 3 Laboratory Medicine Unit, University Hospital of Padova, Padova, 35128 Italy

**Keywords:** Copeptin, Arginine test, Diabetes insipidus, Vasopressin deficiency, Primary polydipsia

## Abstract

**Context:**

A recent multicenter trial confirmed that hypertonic saline-stimulated copeptin is superior to the arginine stimulation test (AST) for diagnosing vasopressin deficiency (AVP-D). The latter, though less accurate, is cheaper, better tolerated, and easier to perform. We aimed to improve AST diagnostic accuracy by incorporating additional parameters alongside copeptin.

**Methods:**

We retrospectively analysed ASTs from patients evaluated for suspected AVP-D. Final diagnosis was defined based on clinical, biochemical, radiological and follow-up data. We evaluated the test diagnostic accuracy based on either literature reported or ROC-based thresholds of several variables even in combination.

**Results:**

Nineteen patients were included and 8 were diagnosed with AVP-D. Copeptin response to AST was flat in AVP-D compared to primary polydipsia (PP) but showed limited discriminatory power with the maximal accuracy for copeptin-based parameters reaching 73.7%. AVP-D patients had lower urinary osmolarity (UOsm) and higher plasma osmolarity and serum sodium (Na) at AST end. Na at AST end was the best predictor of AVP-D (≥ 141 mmol/L: sensitivity 87.5%, specificity 100%, accuracy 94.7%, AUC 0.989). A multistep approach initially assessing Na at AST end and, in dubious cases (140–142 mmol/l), also either copeptin peak (≤ 4.1 pmol/L), UOsm (≤ 428 mOsm/kg), or absent posterior pituitary hyperintense signal achieved 100% diagnostic accuracy. Logistic regression using Na at AST end values combined with any of these aforementioned additional variables also reached complete discrimination between AVP-D and PP.

**Discussion:**

Combining multiple parameters after AST improved diagnostic accuracy, even without measuring copeptin. Despite the study’s retrospective design, small sample, and absence of hypertonic saline testing, findings support the potential utility of a multivariable approach to AST interpretation.

## Introduction

Polyuria polydipsia syndrome with low urinary osmolarity (UOsm) can result from primary polydipsia (PP) [1] or diabetes insipidus; the latter may be caused by either arginine vasopressin deficiency (AVP-D) or resistance (AVP-R) [2]. Diabetes insipidus may have various causes, both congenital and acquired [3]. Copeptin is a stable and easy-to-measure surrogate marker for AVP levels which can aid in making a more accurate diagnosis [4, 5]. In the presence of polyuria, elevated unstimulated copeptin levels (> 21.4 pmol/L) can effectively identify AVP-R [6, 7]. Differential diagnosis between AVP-D and PP may be straightforward based on abnormal sodium (Na) and/or plasma osmolarity (POsm) [8–10]: hyponatremia (< 135 mmol/L) or low POsm (< 280 mOsm/kg) strongly suggest PP, whereas hypernatremia (≥ 147 mmol/L) or elevated POsm (> 300 mOsm/kg) indicate AVP-D. Of note, following pituitary surgery, Na levels of 145–147 mmol/L or higher are generally sufficient for AVP-D diagnosis [11]. However, many patients present with intermediate Na values, requiring additional testing. In recent years copeptin based tests, namely the hypertonic saline test [7, 12] and the arginine stimulation test (AST) [13] have been proposed to differentiate AVP-D and PP with reported better diagnostic accuracy compared to the water deprivation test (WDT). The former is based on an osmotic trigger to the AVP secretion, while the latter relies on arginine as a non-osmotic stimulus, the mechanisms of which are not fully elucidated yet [14]. Glucagon stimulated copeptin also showed promising results [15], but studies on large cohorts are missing to date. A recent head-to-head multicenter trial demonstrated the superiority of hypertonic saline-stimulated copeptin over the AST in diagnosing AVP-D [16]. Although these data point towards the hypertonic saline as the current gold standard to diagnose AVP-D, this test has notable drawbacks, including the requirement for rapid sodium monitoring, medical supervision and stringent safety measures to avoid adverse effects such as seizures. Conversely, arginine-stimulated copeptin is easier to perform, less costly and better tolerated. Consequently, it has been suggested that the AST could serve as a potential first-line diagnostic tool, providing accurate diagnoses (specificity > 90%) for copeptin values ≤ 3 pmol/L indicating AVP-D and for values > 5.2 pmol/L identifying PP. Thus, hypertonic saline test would be necessary only in case of copeptin concentrations between these values [10]. Of note, these novel cut-offs for arginine-stimulated copeptin have not been validated in other cohorts yet.

Our study aims to further evaluate these newly proposed copeptin thresholds for the AST and to identify other parameters that could enhance its performance.

## Methods

### Population

This retrospective study performed at the University Hospital of Padova included patients with non-osmotic polyuria polydipsia syndrome who underwent an AST. We considered patients older than 14 years at first evaluation, with reported urinary output > 50 mL/kg/die and water intake > 3 L daily, with UOsm < 800 mOsm/kg and Na value between 135 and 147 mmol/L (or 145 mmol/L in the postoperative setting), that underwent an arginine-stimulated copeptin test. We excluded patients with rapidly resolving transitory AVP-D following pituitary surgery, patients on diuretics, or those with any of the following: AVP-R, uncontrolled diabetes mellitus, kidney insufficiency, nephrotic syndrome, hypercalcemia, as well as those without sufficient follow-up data to define final diagnosis (see below). Final diagnosis was defined by the authors after reassessment of clinical, biochemical and radiological findings and of follow-up data including the response to treatments (e.g., psychological support, behavioural therapy, low dose desmopressin trial). Participants provided informed consent for the study (PITACORA, protocol number AOP3318, Ethic Committee registration 5938-AO-24).

### Protocols

The WDT protocol depended on symptoms severity. In mild cases (diuresis < 70 mL/kg daily) water restriction began at midnight, with the test starting at 8 a.m.; for ambulatory patients water restriction was not supervised at home and direct observation started at the arrival at our outpatient clinic. Moderate/severe cases were admitted at our inpatient facility the day before test; supervised water restriction started at 6.a.m. and test began at 8 a.m. Weight, blood pressure, urine output and UOsm were measured hourly, while Na and POsm were evaluated every two hours. Maximum duration of the test was 6 h, corresponding to 8 h restriction in moderate/severe cases and 14 h in mild cases. Main criteria for earlier termination were hypotension, hypernatremia (Na > 147 mmol/L) or weight loss > 3%. Concomitant progressive reduction in urine output and increase of UOsm also prompted an early termination [6, 17]. Sodium was determined with ion selective electrode on Cobas system (Roche). The declared repeatabilities (intra-assay imprecisions) were 0.4%, 0.3% and 0.3% for low (88.7 mmol/L), medium (120.6 mmol/L) and high (175.8 mmol/L) levels of sodium, respectively. At the same concentrations, the declared intermediate imprecisions were 1.1%, 0.7% and 0.6%. The within laboratory intermediate imprecisions (during a period of 9 months, *n* = 5497) obtained on 3 instruments, for a total of 5 ion-selective electrodes modules, were 0.8% and 0.6% at 111 mmol/L and 140 mmol/L, respectively. Copeptin was measured at the start and at the end of the WDT with immunofluorescence assay (BRAHMS Copeptin proAVP KRYPTOR, Thermo Fisher Scientific) [6]. According to manufacturer, the limit of quantification was 1.1 pmol/L with intra and inter assay imprecisions range between 1.4–10.7% and 4.7–17.5%, respectively and a reference range < 12.5 pmol/L. Interpretation of the outcome of the WDT was based on UOsm at the end of the WDT and its response to IV desmopressin as previously described [17]. Briefly, UOsm at the end of the WDT above 800 mOsm/kg identified PP, while a value below 300 mOsm/kg indicated complete diabetes insipidus. Among patients with complete diabetes insipidus, AVP-D cases showed a UOsm increase > 50% after 4 mcg of desmopressin IV, as opposed to AVP-R patients. In cases with intermediate UOsm values (300–800 mOsm/kg) at the end of the WDT, following 4 mcg of desmopressin IV, an increase in UOsm greater than 50% was considered consistent with complete AVP-D, a 9–50% increase with partial AVP-D, and a < 9% increase with PP.

AST was performed as follows: after 2 h of thirsting patients received a 0.5 g/kg of arginine 21% (with a maximum dosage of 40 g) administered IV diluted in 500 cc of isotonic saline (0.9%) in 30 min. Copeptin levels were assessed prior to the stimulus and 30, 45, 60, 90 and 120 min after the infusion started [13]. At baseline (prior to arginine infusion) and at 120 min (i.e., at the end of the AST), Na, POsm and UOsm were also assessed.

### Statistical analysis

Categorical variables were reported as counts or percentages, and quantitative variables as median and interquartile ranges [IQR]. The comparisons between independent groups were performed with non-parametric tests, namely a Mann-Whitney sum rank test for quantitative variables and a chi-square test for categorical ones. We used receiving-operator characteristics (ROC) curves to assess diagnostic performance of the variable at various cut-offs and the best performing threshold was defined with the *coords* function (coordinates of a ROC curve), based on Youden’s index. Confidence intervals (CIs) for sensitivity, specificity and diagnostic accuracy were estimated with the Wilson method. The areas under the curves of the ROC curves (ROC-AUC) were compared via *roc.test* function (compare two ROC curves) available in R and the CIs were computed with the default DeLong method. A Fisher exact test was used to compare diagnostic accuracies. Combination of multiple parameters to predict final diagnosis was performed using a logistic regression model, computed with the *glm* function (generalized linear models) available in R. The model provided a linear function, based on the selected variables, to calculate probability distribution (Y) predicting the outcome. As probability distribution is not suitable for clinical practice, we derived from the model’s formula an adjusted value of one predicting variable:

$$\:\text{Y}=\left({\upalpha\:}\text{*}\text{V}1\right)+\left({\upbeta\:}\text{*}\text{V}2\right)+\text{i}\text{n}\text{t}\text{e}\text{r}\text{c}\text{e}\text{p}\text{t}$$

$$\begin{aligned}\:\text{A}\text{d}\text{j}\text{u}\text{s}\text{t}\text{e}\text{d}\:\text{V}1&=\left(\text{Y}-\text{i}\text{n}\text{t}\text{e}\text{r}\text{c}\text{e}\text{p}\text{t}\right)/{\upalpha\:}\\&=\text{V}1+\left[\left({\upbeta\:}/{\upalpha\:}\right)\text{*}\text{V}2\right]\end{aligned}$$

Y is the probability distribution of the linear regression model, V1 and V2 are the predicting variables, α and β are the coefficients of the linear model. The ratio β/α was then assigned a model-specific constant value (k). The AUC for the copeptin response was calculated with the trapezoid formula [18]. Significance threshold was set at p-value < 0.05. Statistical analyses were performed with R (R Core Team (2022). R: A language and environment for statistical computing. R Foundation for Statistical Computing, Vienna, Austria. URL https://www.R-project.org/) and R studio (Posit team (2024). RStudio: Integrated Development Environment for R. Posit Software, PBC, Boston, MA. URL http://www.posit.co/).

## Results

Nineteen patients who underwent an AST at our center between 2019 and 2024 were included. Ten of them underwent also a WDT in either the inpatient or outpatient setting (see methods). Most patients were female (12/19, 63%), with a median age at the time of the test of 38 years [24; 50]. Main reason for testing was the clinical suspicion (i.e., polyuria polydipsia syndrome), while in 5 (26%) cases there was a known pituitary disease/intervention. Eight patients were finally diagnosed as AVP-D (42%) from various causes (2 Langerhans cell histiocytosis, 1 post-traumatic, 1 prolactinoma apoplexy, 1 pituitary xanthogranuloma, 1 following non-functioning pituitary adenoma surgery, 2 idiopathic). Estimated diuresis at home ranged from 3 to 8.5 L per day, with a median of 4.0 L [3.5; 5.5]. Information regarding the posterior pituitary hyperintense signal in T1 images (PPHS) on MRI [19] was available for 16 patients.

### Comparison between water deprivation test and arginine-stimulated copeptin

Ten patients underwent both WDT and AST, with 3 receiving a final diagnosis of AVP-D. When applying both the classical 60 min copeptin cut-off of 3.8 pmol/L [13] and the more recently proposed threshold of 4.1 pmol/L at 90 min [16], the arginine-stimulated copeptin appeared less accurate than the WDT (Table 1); the difference was not statistically significant due to the small sample (*p* = 0.3). Most WDTs were stopped early: in 5 cases there was diuresis contraction paired with progressive UOsm raise, three patients developed hypernatremia, a patient experienced hypotension. When comparing AVP-D and PP patients, copeptin values at the beginning (2.1 [1.8; 2.6] vs. 1.8 [1.4; 2.9], *p* = 0.71) and at the end (1.9 [1.8; 2.5] vs. 2.0 [1.8; 3.1], *p* = 0.52) of the WDT did not differ. No patient had a copeptin above 4 pmol/L at the end of the WDT [20].

**Table 1:** Performance of arginine-stimulated copeptin and water deprivation test in the diagnosis of vasopressin deficiency (AVP-D) in 10 patients. CI: confidence interval; m: minutes

Test	Cut-off for AVP-D	Sensitivity (95%CI)	Specificity (95%CI)	Diagnostic accuracy (95%CI)
Arginine-stimulated copeptin	Copeptin 60 m ≤ 3.8 pmol/L	100% (43.8–100)	42.9% (15.8–75)	60.0% (31.3–83.2)
Arginine-stimulated copeptin	Copeptin 90 m ≤ 4.1 pmol/L	100% (43.8–100)	42.9% (15.8–75)	60.0% (31.3–83.2)
Water deprivation test	See methods	100% (43.8–100)	85.7% (48.7–97.4)	90.0% (59.6–98.2)

### Arginine stimulation test

**Fig. 1:**
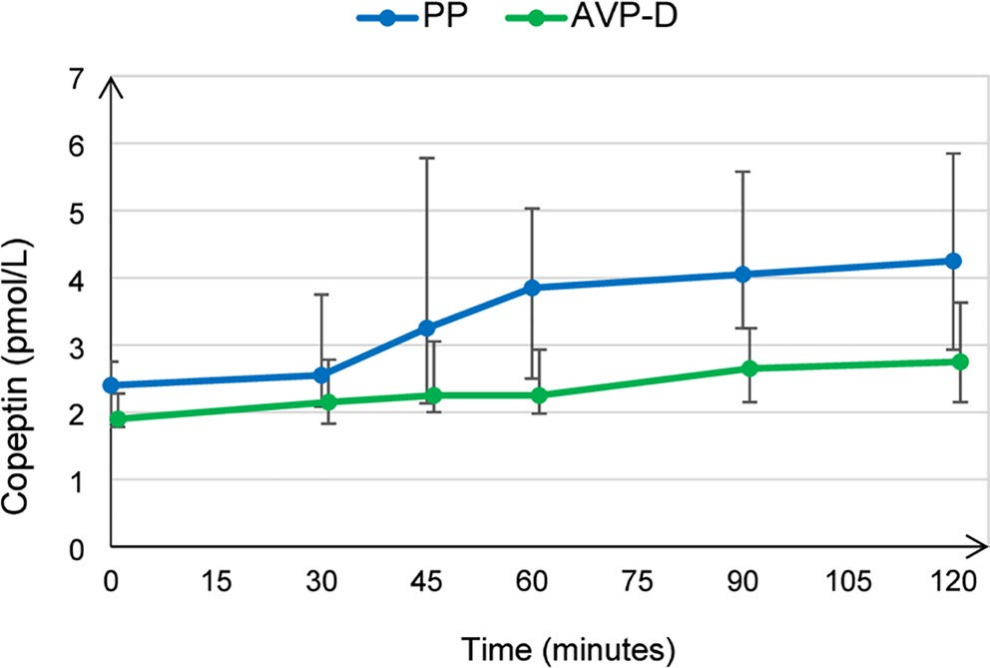
Median copeptin values following arginine stimulation in arginine-vasopressin deficiency (AVP-D) and primary polydipsia (PP); interquartile range is reported at each time point for both groups

When comparing the two groups, patients with AVP-D showed higher Na and POsm both at baseline and at the end of the AST; UOsm at the end of the AST was also significantly lower and the absence of PPHS was more frequent in AVP-D as expected. AVP-D patients tended to be older and exhibited a flat response to arginine (Table 2; Fig. 1). No patient reported any adverse events following AST. Notably, three patients had a weight above 80 kg and received the maximum arginine dose of 40 g IV.

**Table 2:** Comparison of multiple parameters between patients with vasopressin deficiency and primary polydipsia. Interquartile ranges are reported within the brackets. Na: serum sodium; POsm: plasma osmolarity; UOsm: urinary osmolarity; m: minutes of the arginine stimulation test; PPHS: posterior pituitary hyperintense signal in T1 images

Feature	Primary polydipsia	Vasopressin deficiency	*p*
**Age** (years)	32 [24–39]	43 [39–55]	*0.09*
**Female sex**	6/8 (75%)	6/11 (55%)	0.67
**Urinary output** (L per day)	4.0 [3.6–5.5]	4.0 [3.4–5.8]	0.87
**Na 0 m** (mmol/L)	140 [138–141]	144 [143–146]	**< 0.01**
**POsm 0 m** (mOsm/kg)	282 [277–285]	289 [288–295]	**< 0.01**
**UOsm 0 m** (mOsm/kg)	319 [220–541]	185 [141–281]	0.18
**Na 120 m** (mmol/L)	139 [138–140]	145 [142–148]	**< 0.01**
**POsm 120 m** (mOsm/kg)	289 [287–293]	299 [297–307]	**< 0.01**
**UOsm 120 m** (mOsm/kg)	463 [404–560]	248 [159; 303]	**< 0.01**
**Copeptin 0 m** (pmol/L)	2.4 [2.0–2.8]	1.9 [1.8–2.3]	0.24
**Copeptin 30 m** (pmol/L)	2.6 [2.1–3.8]	2.2 [1.8–2.8]	0.23
**Copeptin 45 m** (pmol/L)	3.3 [2.1–5.8]	2.3 [2.0–3.1]	0.17
**Copeptin 60 m** (pmol/L)	3.9 [2.5–5.0]	2.3 [2.0–2.9]	*0.08*
**Copeptin 90 m** (pmol/L)	4.1 [3.3–5.6]	2.7 [2.2–3.3]	*0.05*
**Copeptin 120 m** (pmol/L)	4.3 [2.9–5.9]	2.8 [2.2–3.6]	*0.08*
**Copeptin peak** (pmol/L)	4.3 [3.3–6.1]	2.9 [2.3–3.6]	*0.06*
**Copeptin AUC** (pmol*h/L)	7.0 [5.4–9.5]	4.5 [4.2–6.1]	0.11
**Presence of PPHS**	6/8 (75%)	2/11 (18%)	**0.04**

Besides the predefined thresholds from literature (i.e., copeptin 3.8 pmol/L at 60 min and 4,1 pmol/l at 90 min), we assessed also ROC-based cut-offs for other parameters. Various copeptin-related parameters (0, 60, 90 and 120 min, peak and AUC) demonstrated similar diagnostic accuracy with ROC-based thresholds; the 90 min copeptin value provided a slightly higher ROC-AUC (Table 3). Considering the above-mentioned copeptin thresholds of 3 and 5.2 pmol/L, two patients were misdiagnosed. A copeptin peak above 5.2 pmol/L correctly identified 4/4 (100%) patients with PP, while values ≤ 3 pmol/L correctly diagnosed AVP-D only in 5/7 patients (71%). In our cohort 8 patients presented with intermediate copeptin values. Nonetheless, indirect parameters (namely Na, POsm an UOsm) at the end of the AST provided greater diagnostic accuracy than copeptin measures discussed above, especially Na levels (Table 3).

**Table 3:** Performance of various cut-offs for diagnosing vasopressin deficiency (AVP-D) via arginine stimulation test (AST). * ROC-AUC from sodium level at the end of the AST was significantly higher than the ones derived from copeptin AUC and copeptin at 60 and 120 min (*p* < 0.05), while other comparisons were not significantly different. ROC: receiving operator characteristics curve; CI: confidence interval; AUC: area under the curve; Na: serum sodium; POsm: plasma osmolarity; UOsm: urinary osmolarity; m: minutes; n.a.: not available

Parameter	Cut-off for AVP-D	Type	Sensitivity (95%CI)	Specificity (95%CI)	Diagnostic accuracy (95%CI)	ROC-AUC* (95%CI)
Copeptin 60 m	≤ 3.8 pmol/L	Predefined (13)	100% (67.6–100)	54.6% (28.0–78.7)	73.7% (51.2–88.2)	n.a.
Copeptin 90 m	≤ 4.1 pmol/L	Predefined (16)	87.5% (52.9–97.8)	54.6% (28.0–78.7)	68.4% (46.0–84.6)	n.a.
Copeptin 0 m	≤ 2.1 pmol/L	ROC-based	75% (40.9–92.9)	72.7% (43,4–90.2)	73.7% (51.2–88.2)	0.665 (0.398–0.932)
Copeptin 30 m	≤ 3.5 pmol/L	ROC-based	100% (67.6–100)	45.5% (21.3–72.0)	68.4% (46.0–84.6)	0.671 (0.416–0.925)
Copeptin 60 m	≤ 3.9 pmol/L	ROC-based	100% (67.6–100)	54.6% (28.0–78.7)	73.7% (51.2–88.2)	0.744 (0.508–0.981)
Copeptin 90 m	≤ 3.5 pmol/L	ROC-based	87.5% (52.9–97.8)	63.6% (35.4–84.8)	73.7% (51.2–88.2)	0.773 (0.547–0.998)
Copeptin 120 m	≤ 3.9 pmol/L	ROC-based	87.5% (52.9–97.8)	63.6% (35.4–84.8)	73.7% (51.2–88.2)	0.750 (0.523–0.977)
Copeptin peak	≤ 4.6 pmol/L	ROC-based	100% (67.6–100)	54.6% (28.0–78.7)	73.7% (51.2–88.2)	0.767 (0.544–0.991)
Copeptin AUC	≤ 7.9 pmol*h/L	ROC-based	100% (67.6–100)	54.6% (28.0–78.7)	73.7% (51.2–88.2)	0.727 (0.484–0.971)
Na 0 m	≥ 142 mmol/L	ROC-based	75% (40.9–92.9)	100% (74.1–100)	89.5% (68.6–97.1)	0.932 (0.925–1.0)
Na 120 m	≥ 141 mmol/L	ROC-based	87.5% (52.9–97.8)	100% (74.1–100)	94.7% (75.4–99.1)	0.989 (0.962–1.0)
POsm 0 m	≥ 287 mOsm/kg	ROC-based	87.5% (52.9–97.8)	81.8% (52.3–94.9)	84.2% (62.4–94.5)	0.903 (0.772–1.0)
POsm 120 m	≥ 294 mOsm/kg	ROC-based	87.5% (52.9–97.8)	90.9% (62.3–98.4)	89.5% (68.6–97.1)	0.915 (0.756–1.0)
UOsm 120 m	≤ 277 mOsm/kg	ROC-based	75% (40.9–92.9)	90.9% (62.3–98.4)	84.2% (62.4–94.5)	0.886 (0.737–1.0)

**Fig. 2:**
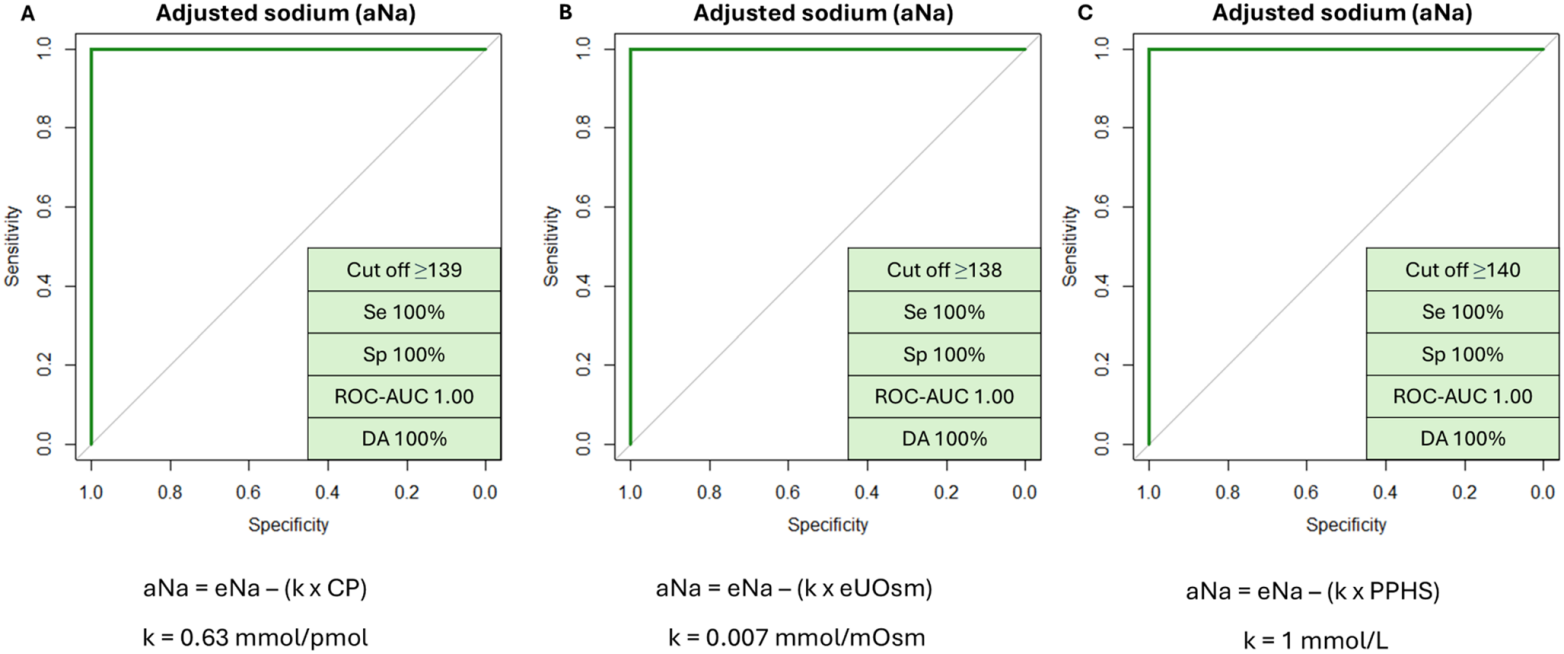
ROC curves based on adjusted sodium value (aNa) at the end of the arginine stimulation test (AST) for diagnosing vasopressin deficiency. Three different models were used, combining sodium at the end of the AST (eNa) with (**A**) copeptin peak (CP), (**B**) urinary osmolarity at the end of the AST (eUOsm) or (**C**) presence of posterior pituitary hyperintense signal in T1 images (PPHS). Note that, based on the presence or absence of the posterior pituitary hyperintense signal in T1 images, PPHS is assigned a value of 1 or 0, respectively. ROC-AUC was not significantly different from that provided by eNa alone (*p* = 0.41). AUC: area under the curve; Se: sensitivity; Sp: specificity; DA: diagnostic accuracy; k: model-specific coefficient

We tried to enhance the performance of the AST by also considering multiple parameters, the best performing of which (i.e., Na at 120 min, namely at the end of the AST – eNa) was selected and combined with other variables through logistic regression models. Among available copeptin-related variables, we chose the copeptin peak. We also considered other variables that significantly differed between AVP-D and PP, namely UOsm at 120 min (i.e., at the end of the AST – eUOsm) and the presence of PPHS. POsm was not included as it is expected to closely correlate with serum Na values. The resulting regression model with each combination provided a complete discrimination between AVP-D and PP (ROC-AUC 1.00). We tried to simplify the model to ease its application in clinical practice by deriving a formula for an adjusted Na value (aNa) (see methods). aNa was defined as follows: aNa = eNa – (k*V2), with eNa being the sodium value (measured in mmol/L) at the end of the AST, V2 being the second considered variable and k a model-specific coefficient derived from the linear regression model (Table 4). Using aNa allowed complete discrimination with each second variable considered (Fig. 2).

**Table 4:** Logistic regression models created with glm function (see methods) and derivation of an adjusted sodium value (aNa): sodium value at the end of the arginine stimulation test (eNa) was combined with a second predicting variable (V2). * Coefficients and intercepts have been approximated. ** PPHS = 1 if the signal is present and PPHS = 0 if the signal is absent

V2	Linear regression model*	Derived formula for adjusted Na (aNa)	k value	aNa cut-off for AVP-D
Copeptin peak after arginine stimulation (CP)	Y = (24.0*eNa)– (15.2*CP) – 3336.7	aNa = (Y + 3336.7)/24 = = eNa – (0.63*CP)	0.63 mmol/pmol	≥ 139 mmol/L
Urinary osmolarity at the end of arginine stimulation test (eUOsm)	Y = (90.9*eNa)– (0.7*eUOsm) – 12515.2	aNa = (Y + 12515.2)/90.9 = = eNa – (0.007*eUOsm)	0.007 mmol/mOsm	≥ 138 mmol/L
Presence of posterior pituitary hyperintense signal in T1 images** (PPHS)	Y = (43.6*eNa)– (43.6*PPHS) – 6122.6	aNa=(Y + 6122.6)/43.6 = = eNa – (1*PPHS)	1 mmol/L	≥ 140 mmol/L

**Fig. 3:**
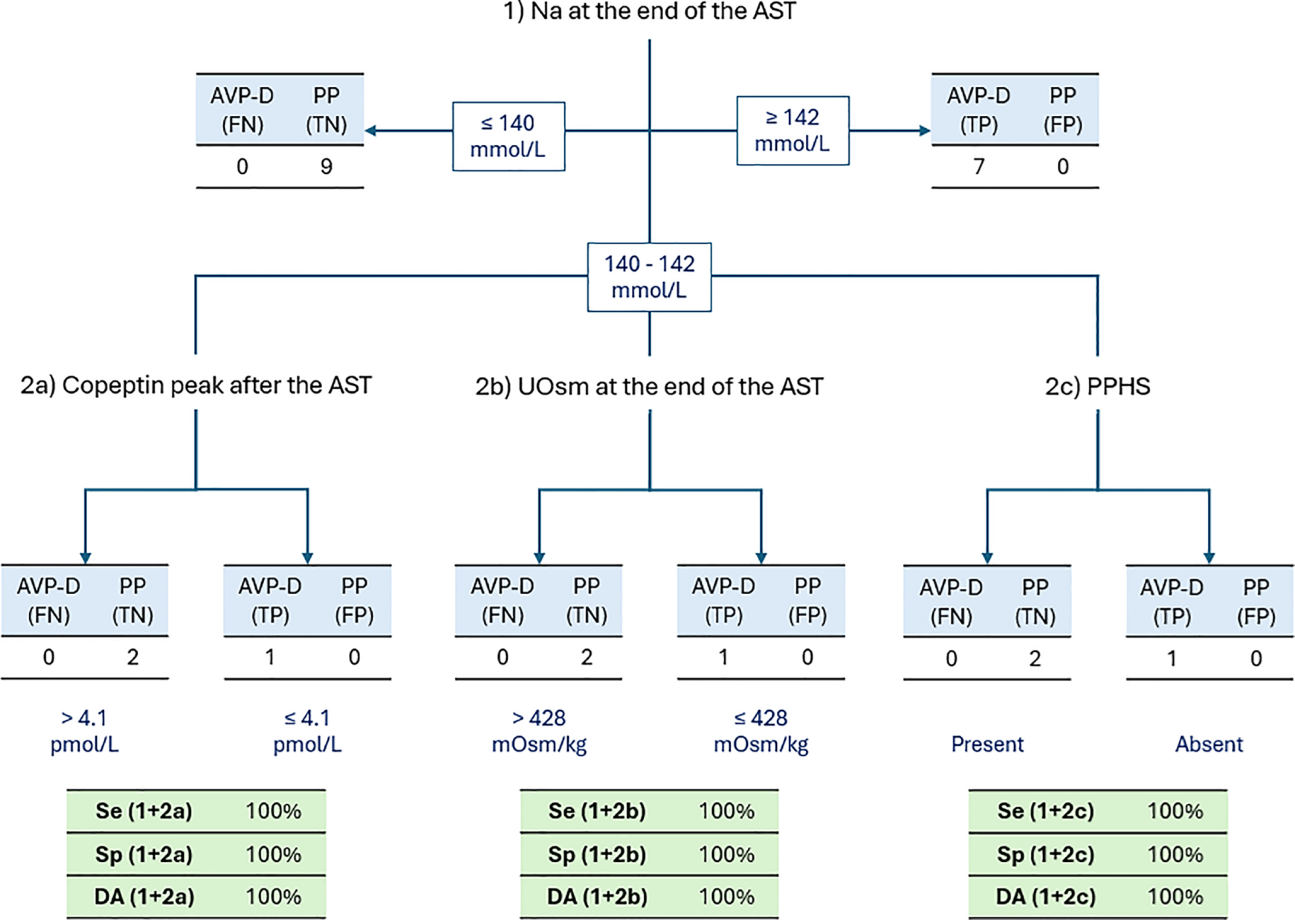
Multistep diagnostic process comprising of a first step evaluating serum sodium (Na) at the end of the arginine stimulation test (AST) and a second step evaluating either copeptin peak after the AST, urinary osmolarity (UOsm) at the end of the AST or the presence of posterior pituitary hyperintense signal in T1 images (PPHS). AVP-D: arginine-vasopressin deficiency; PP: primary polydipsia; Se: sensitivity; Sp: specificity; DA: diagnostic accuracy

Another possible approach was to apply a multistep process. We first defined the thresholds for Na value at the end of AST that allowed 100% specificity and 100% sensitivity respectively, namely ≤ 140 mmol/l consistent with PP and ≥ 142 mmol/L consistent with AVP-D. In case of intermediate values, further parameters were used to complete the differential diagnosis: a copeptin peak after the AST ≤ 4.1 pmol/L, a UOsm at the end of the AST ≤ 428 mOsm/kg or the absence of PPHS identified AVP-D (Fig. 3). All combinations allowed a complete discrimination between AVP-D and PP (diagnostic accuracy 100%, ROC-AUC 1.00).

## Discussion

The differential diagnosis between AVP-D and PP in cases of intermediate, non-diagnostic sodium values and non-elevated basal copeptin levels may be challenging. The recent paper from Refardt and collaborators pointed at the hypertonic saline-stimulated copeptin as the gold standard test for identifying AVP-D, on the basis of its higher diagnostic accuracy (95.6%, 95% CI [91.1–97.8])) compared to the AST (74.4%, 95%CI [67.0–80.6]) [16]. These data suggest a shift in the current diagnostic paradigm by emphasizing the role of hypertonic saline-stimulated copeptin. The final diagnosis in that study partly relied on the results of the hypertonic saline–stimulated copeptin test, whilst AST results were not considered, introducing a risk of bias towards higher accuracy of the former (although this incorporation bias was mitigated by the critical appraisal of the results based on global picture and follow-up data). Arginine-stimulation nevertheless offers various advantages as it is less costly and shorter, can be performed in the outpatient setting, does not require constant medical supervision and rapid sodium measurements for safety, is better tolerated, and has no specific contraindications. Given these practical advantages, investigators have been able to improve the specificity and/or sensitivity of the AST by adopting multiple cut-offs: a stimulated peak serum copeptin ≤ 3.0 pmol/L correctly identified AVP-D in 90.9% of cases, whereas a response > 5.2 pmol/L diagnosed true PP in 91.4% of cases. Therefore, it was suggested that the AST could be used as an initial procedure followed by hypertonic saline stimulation in case of intermediate copeptin levels (3.0–5.2 pmol/L) [10].

Although the above-mentioned diagnostic process is undoubtedly feasible, enhancing the performance of the arginine-stimulated copeptin could reduce the need for a hypertonic saline-stimulated test. In our cohort we confirmed a suboptimal performance of arginine-stimulated copeptin parameters, with the same diagnostic accuracy (73.7%, 95%CI [52.6–89.5]) observed across several parameters (0, 60, 90 and 120 min, peak and AUC). In fact, the use of previously identified cut-off values of ≤ 3.0 pmol/L for AVP-D and > 5.2 pmol/L for PP, still misdiagnosed 2/11 patients (18%) in our cohort.

Interestingly, indirect parameters (Na, POsm, UOsm) obtained at the end of the AST provided a better diagnostic performance than copeptin related measures, particularly Na at 120 min (diagnostic accuracy 94.7%, 95%CI [75.4–99.1]). Serum Na levels significantly differed between AVP-D and PP even at the beginning of the test, prior to arginine stimulation and after an initial thirsting period of at least 2 h (*p* < 0.01). In fact, Na could be influenced by both osmotic and non-osmotic (via arginine stimulation) factors. In our series, Na values at the beginning and at the end of the AST did not significantly differ in each of the 2 groups. It is difficult to interpret this lack of change of serum Na levels between 0 and 120 min given the interaction of multiple factors affecting Na levels in possibly either direction, namely oral fluid restriction, 500 cc of IV 0.9% saline, and pharmacological effects of arginine. Nevertheless, since all these three are part of the AST protocol, the potential of Na value to improve test accuracy remains, as long as the test is standardized. If confirmed in larger series, the use of indirect parameters may not only provide a novel approach to enhance the AST performance but also expand its applicability. In fact, copeptin measurement is still not widely available, while sodium sampling is nowadays part of the biochemical routine worldwide.

We also tried to combine multiple parameters to improve the performance of the AST. Interestingly, both a multistep approach and a formula for an adjusted sodium value (aNa) achieved complete discrimination between AVP-D and PP at the end of the AST. Surprisingly, this could be obtained without copeptin measurements, by combining Na at the end of the AST with either UOsm at the end of the AST or imaging information. In line with prior reports, PPHS confirmed its limited utility in the diagnosis of AVP-D when considered alone (Table 1) [19].

Copeptin > 4.0 pmol/L following overnight water deprivation (after 8 h of thirsting) was recently deemed effective by another group [20], in ruling out AVP-D. However, in our cohort, at the end of the WDT, no patient had a copeptin level above 4 pmol/L and copeptin values did not differ between AVP-D and PP. Notably, in 3 out of 4 patients with urinary output > 5 L daily who underwent inpatient WDT starting at 6 a.m. the test was stopped early, resulting in a water deprivation of less than 8 h: 4 h in one AVP-D case due to hypernatremia and 7 h in two PP patients due to hypotension and UOsm raise respectively (copeptin values at the end of the WDT were 1.3 and 2.1 pmol/l). Although unlikely, the possibility of copeptin raising above 4 pmol/l with an additional hour of thirsting in the two PP patients cannot be ruled out. Still, the possibility of relying on copeptin values after the WDT for distinguishing between AVP-D and PP cannot be supported by our experience. On the other hand, classical criteria of the WDT performed well, with a diagnostic accuracy of 90%.

Similarly, copeptin levels prior to arginine stimulation did not differ between AVP-D and PP patients (*p* = 0.24) with resulting suboptimal diagnostic accuracy (Tables 2, 3).

This study undoubtedly has several limitations, with the most relevant being the small sample size, its retrospective nature and the lack of hypertonic saline-stimulation test to validate the final diagnosis. In line with previous studies, results of the tests (WDT and AST) were also considered in defining the final diagnosis of AVP-D, although they were critically evaluated based on the overall clinical picture, disease course during follow-up and response to treatment. Moreover, serum Na range at the end of the AST, on which we based our regression models (Fig. 2) and diagnostic flowchart (Fig. 3), may not be exactly reproducible in other centers or situations, such as concomitant medical conditions and different local instrument calibration/reference ranges. Finally, the arginine dose used in the three overweight patients might have been a suboptimal stimulus for copeptin response.

Clinical work-up in AVP-D diagnosis is well supported by evidence from large multicenter prospective trials. Far from changing the current reference standard, our limited experience with the AST suggests that a more comprehensive approach could enhance diagnostic accuracy and broaden the applicability of this easy and safe protocol. Future prospective studies with larger cohorts and the use of current gold standard test to define AVP-D (i.e., hypertonic saline test) are needed to evaluate whether our approach to AST interpretation could have a more generalized application in clinical practice.

## Data Availability

All data underlying the results are available as part of the article and no additional source data is required.
